# Cognitive Performance in Older Adults With Subjective Cognitive Decline

**DOI:** 10.1093/geroni/igaa057.932

**Published:** 2020-12-16

**Authors:** Vanessa Taler, Cassandra Morrison, Christine Sheppard

**Affiliations:** 1 University of Ottawa, Ottawa, Ontario, Canada; 2 Sunnybrook Research Institute, Toronto, Ontario, Canada

## Abstract

Subjective cognitive decline (SCD) refers to a perceived decline in cognitive function in the absence of neuropsychological deficits. Older adults with SCD are at increased of subsequent development of mild cognitive impairment or dementia. We had 224 adults aged 65+ complete questionnaires assessing their subjective appraisal of their cognitive function, including questions about word-finding difficulty, memory, and attention/concentration. Participants also completed the Montreal Cognitive Assessment (MoCA). All participants exhibited cognitive performance that was within normal limits for age and education. In total, 29.5% of participants reported word-finding difficulties, 16.5% reported difficulties with remembering things, and 8.5% reported difficulties with attention/concentration. We found that (1) self-reported word-finding difficulties were associated with lower performance on delayed word recall, and (2) self-reported difficulties in concentration/attention or memory were associated with lower performance on the abstraction subtask in the MoCA. No other MoCA subtasks were associated with self-reported cognitive function. A subset of the participants (n=69) also completed a battery of tasks assessing semantic function, including picture naming, associative matching tasks, identification of semantic features, and semantic questions. Again, self-reported word-finding difficulty predicted lower performance on semantic tasks. These results suggest that older adults may be aware of changes in their cognitive performance prior to objective neuropsychological impairment. Moreover, their awareness appears to be domain-specific: self-reported language difficulty is associated with lower performance on language-based tasks, while self-reported difficulty in memory, attention, or concentration is associated with lower performance on an abstraction task.

